# Blood donation and subjective wellbeing: a cross-sectional survey and a randomized trial

**DOI:** 10.3389/fpsyg.2026.1795243

**Published:** 2026-03-27

**Authors:** Jian Ou-Yang, Fan-fan Feng, Rong-rong Ke, Xiao-ying Huang, Hua-qin Liang, Jin-yan Chen

**Affiliations:** 1 Guangzhou Blood Center, Guangzhou, Guangdong, China; 2 The Key Medical Laboratory of Guangzhou, Guangzhou, Guangdong, China

**Keywords:** basic psychological needs, blood donation, prosocial behavior, self-determination theory, subjective wellbeing

## Abstract

**Introduction:**

Prosocial behavior has been widely associated with higher subjective wellbeing (SWB), yet little is known about whether blood donation, a distinct type of formal prosocial behavior, produces similar psychological benefits. Applied Self-Determination Theory, this study examined the relationship between blood donation and SWB, and explored whether satisfaction of basic psychological needs (BPN; autonomy, competence, and relatedness) explains this association. Understanding these psychological benefits may also provide insights for improving blood donor recruitment and retention.

**Methods:**

A two-phase design was employed. Phase 1 was a cross-sectional survey comparing 601 whole blood donors (Donation group) with 1,043 non-donors, including participants recalling prosocial acts (Recall group, *n* = 517) and controls with no recall task (Non-recall group, *n* = 526). Phase 2 was a single-center randomized trial in which donors were allocated to receive follow-up questionnaires either with (Intervention group, *n* = 301) or without (Control group, *n* = 300) a gratitude reinforcement message highlighting their donation’s life-saving impact. SWB was assessed using measures of positive affect, negative affect, and life satisfaction. BPN satisfaction was measured using validated scales. ANCOVA models were used to estimate intervention effects while controlling for baseline levels.

**Results:**

Cross-sectional analyses indicated that both blood donation and recalling prosocial behavior were positively associated with Time-1 SWB compared with the Non-recall group. Mediation analyses further showed that Time-1 BPN satisfaction significantly mediated the association between blood donation and Time-1 SWB. In the trial, ANCOVA models indicated that the gratitude reinforcement message significantly increased Time-2 BPN satisfaction but was not directly associated with Time-2 SWB. However, mediation analyses suggested an indirect pathway whereby the intervention was associated with higher Time-2 SWB through increased Time-2 BPN satisfaction, particularly competence satisfaction.

**Discussion:**

This research represents a first step in understanding the relationship between blood donation and SWB. Findings suggest that blood donation may be associated with improved wellbeing partly through the satisfaction of BPN. Reinforcing donors’ perceived impact may strengthen these psychological benefits, although stronger feedback interventions may be required to produce measurable improvements in wellbeing. Future research should examine these effects longitudinally and across cultures.

**Clinical trial registration:**

Clinicaltrials.gov, identifier NCT05213130.

## 1 Introduction

In recent decades, a series of theoretical discussions and empirical studies have been carried out on prosocial behaviors and wellbeing. A substantial body of research shows that there is a positive relationship between prosociality and wellbeing (Yinon and Landau, 1987; Brunstein et al., 1996; Cialdini et al., 1997; Schwartz et al., 2003; Hui et al., 2020; Haller et al., 2022). Prosocial behavior is a general concept that refers to action intended to benefit others (Dovidio et al., 2017), which are crucial behaviors that contribute to a harmonious and well-functioning society (Penner et al., 2005). The mechanisms by which acting prosociality may benefit help-givers include increasing self-evaluations and perceived competence, distracting help-givers from their own troubles and stress, enhancing the realization of life’s meaning and value, boosting positive moods, and facilitating social integration (Midlarsky, 1991; Hui et al., 2020). Moreover, this relationship is theorized to be reciprocal, with elevated wellbeing potentially fostering further prosocial engagement, thereby creating a virtuous cycle (Aknin et al., 2012).

Prosocial behaviors—such as helping a stranger, volunteering, donating blood, and giving to charity—are actions that benefit others and society as a whole. An old Chinese proverb states that “Gifts of roses have a lingering fragrance”, which is also a common slogan in blood donation campaigns to highlight the emotional rewards of giving in China. Hinrichs et al. (2008) found that wellbeing was associated with a positive and successful blood donation experience. Their findings suggest blood donation may offer emotional benefits by redirecting attention from daily stressors, though this depends on the donation process being perceived as minimally stressful (Hinrichs et al., 2008). However, relatively little research has examined how blood donation influences donors’ wellbeing, particularly in non-Western contexts. The present study aims to examine whether blood donation can enhance donor’ wellbeing in a Chinese sample, with the ultimate goal of encouraging more individuals to become regular donors.

Blood donation in Mainland China follows national regulations that outline strict eligibility criteria and standardized procedures to ensure donor safety. Individuals aged 18–55 (regular donors who meet health requirements are eligible to continue until the age of 60) who meet hemoglobin, blood pressure, weight, and medical screening requirements may donate whole blood every 180 days, or apheresis platelets every 90 days. According to recent reports from the National Health Commission (Office of the National Health Commission, 2026), China’s voluntary non-remunerated blood donation rate remains relatively modest at approximately 11 donations per 1,000 population in recent years. Although public awareness of donation campaigns has gradually increased, only a small proportion of eligible adults donate regularly, and supply–demand gaps persist in many regions. Cultural perceptions that blood donation may weaken the body further contribute to low participation rates (Chen and Ma, 2015; Ou-Yang et al., 2017). These contextual factors shape the emotional and motivational experience of blood donors and underscore the need for research examining how psychological benefits may support donor engagement in this setting.

Hedonic wellbeing and eudaimonic wellbeing are two important sub-types of wellbeing. Hedonic wellbeing is based on the notion that increased pleasure and decreased pain leads to happiness. One dominant approach in hedonism is to assess subjective wellbeing (SWB) (Diener et al., 2002), which is a self-reported measure of wellbeing. It comprises both an affective component (high positive affect and low negative affect) and a cognitive component (satisfaction with life) (Diener, 1984; Lucas et al., 1996; Diener and Biswas-Diener, 2002; Diener et al., 2003). Blood donation is a specific, context-dependent behavior whose psychological impact is more likely to manifest in emotional experiences and life satisfaction rather than in more abstract dimensions of wellbeing (e.g., meaning in life or social functioning). SWB is therefore suited to capture the immediate and long-term effects of blood donation on individuals.

In recent years, Self-Determination Theory (SDT; Deci and Ryan, 2000) has been used to explain the mechanisms by which prosociality benefits SWB (Hui and Kogan, 2018; Wray-Lake et al., 2019; Hui et al., 2020; Chen, 2024). According to SDT, human wellbeing depends on the satisfaction of three basic psychological needs (BPN): relatedness, competence, and autonomy. SDT provides a comprehensive framework that considers multiple psychological needs and their impact on SWB. This approach is particularly useful for studying complex behaviors like blood donation, which can be influenced by a variety of internal and external factors (France et al., 2017). Applying SDT, we hypothesized that blood donation satisfied blood donors’ need for competence (when they know their blood has saved a patient’s life), need for relatedness (by ensuring future reciprocity, such as the idea of having blood available for relatives in the future) (Ferguson et al., 2020); need for autonomy (when the donation is autonomously motivated). The present study conceptualized the satisfaction of BPN as an integrated construct reflecting overall psychological fulfillment. Although these needs are theoretically distinct, they are highly interrelated and tend to operate jointly in influencing wellbeing (Hui and Kogan, 2018; Deci and Ryan, 2000). Accordingly, we used an overall BPN satisfaction index as the primary mediator to capture the general sense of need satisfaction arising from engaging in prosocial behavior. To ensure robustness, we also examined each need separately in supplementary analyses.

A key methodological challenge in studying the wellbeing benefits of prosocial behavior is separating the effects of merely reflecting on one’s prosociality from those of actually performing a prosocial act. To address this issue, we included a group of non-donors who recalled a past prosocial behavior (Recall group), allowing us to isolate the unique contribution of blood donation itself. While recalling prosocial acts can enhance wellbeing by activating positive self-perceptions and memories of kindness (Ko et al., 2021), blood donation involves additional elements, including physical engagement, participation in a formal and socially meaningful procedure, and the knowledge that one’s blood will directly help save a life. Comparing the Donation and Recall groups therefore provides a more stringent test of whether blood donation confers psychological benefits beyond those elicited by cognitive reflection alone. Then we examined whether wellbeing associated with blood donation was related to subsequent donation intention, assessing whether emotional rewards may support sustained prosocial engagement. Finally, we further examined whether reinforcing the meaning of donation could enhance donors’ wellbeing. Specifically, blood donors were randomly assigned to receive a brief gratitude reinforcement message or no message after donation, allowing us to test whether a simple, low-cost intervention could further amplify the wellbeing benefits of blood donation. We hypothesize that:

*H1*: Both engaging in blood donation (Donation group) and recalling acts of prosocial behavior (Recall group) will demonstrate significantly higher levels of SWB at Time-1 compared to the Non-recall group.

*H2*: The relationship between blood donation and SWB will be mediated by the satisfaction of BPN.

*H3*: Higher levels of SWB at Time-1 will be positively associated with greater blood donation intention in the Donation group.

*H4a*: Blood donors who receive a gratitude reinforcement message emphasizing the impact of their donation (e.g., saving a patient's life) (Intervention group) will report greater improvements in both SWB and BPN satisfaction from Time-1 to Time-2, compared to those who do not receive such a message (Control group). H4b: This effect will be mediated by increased satisfaction of BPN.

All procedures of this study were reviewed and approved by the Institutional Review Board of the Guangzhou Blood Center. The registration ID for randomized trial in this study on ClinicalTrial.gov is NCT05213130. The preregistered components of this trial include: (1) the randomized assignment to the Intervention versus Control condition; (2) the timing of data collection at Time-1 and Time-2; (3) the primary psychological outcomes (SWB at follow-up); and (4) the inclusion and exclusion criteria for blood donors established by the Guangzhou Blood Center. We reported this study according to the Consolidated Standards of Reporting Trials statements (Supplementary material 1).

## 2 Materials and methods

### 2.1 Cross-sectional baseline survey

#### 2.1.1 Study design, setting and participants

To test H1 and H2, a cross-sectional baseline survey was conducted. From January 18 to February 25, 2022, whole blood donors at mobile drives in Guangzhou were invited by Guangzhou Blood Center staff to complete an online questionnaire (Donation group). Two groups of non-donors were recruited via a digital online survey tool called “WEN JUAN XING”,^1^ which is popular in Mainland China and functions similarly to Amazon’s Mechanical Turk. WEN JUAN XING recruited participants from January 28, 2022 to February 10, 2022. Participants aged 18–60 (the legal age range for blood donation in Mainland China) who had never donated blood were eligible. Previous research has demonstrated that COVID-19 containment measures, such as lockdowns and social distancing policies, significantly impact SWB (Bojanowska et al., 2021; Brindal et al., 2022). To control for potential regional variations in COVID-19 containment measures and their differential impacts on wellbeing outcomes across China, we exclusively selected respondents with IP addresses originating from Guangzhou. Non-donors who answered a questionnaire recalling a past prosocial experience were placed in the Recall group, while those who answered the same questionnaire but without being prompted to recall were assigned to the Non-recall group. Blood donors in this stage were voluntary and were not incentivized. Non-donors would receive points by WEN JUAN XING.

Sample size was predetermined based on sample size recommendations (Le et al., 2018), financial considerations, time constraints, and the high attrition rate to attain around 500 valid participants per group. Data validity was assessed using an attention-check item (“This is a test question—please select the number TWO”). The principal investigator monitored responses daily and closed enrolment once the target sample size was reached. In the Donation group, participants could provide their mobile phone number for follow-up if they consented. Only donors who provided contact information and whose blood was successfully processed and supplied to hospitals were included in the final sample.

#### 2.1.2 Baseline questionnaires

After providing informed consent, participants in the Recall group were asked to recall a previous prosocial behavior, whereas those in the Donation and Non-recall groups proceeded directly to the SWB and BPN measures. All scales used in this study were based on previously validated Chinese versions (Xiong and Xu, 2009; Qiu et al., 2008; Xie et al., 2012). To ensure conceptual and linguistic equivalence, these versions had been developed following the widely recommended translation-back-translation procedure (Brislin, 1970). Sociodemographic characteristics, such as gender, current marital status, education, employment status, and household per capita monthly income were collected. Blood donors also provided their donation history (frequency) and rated their intention to donate blood in the future.

##### 2.1.2.1 Subjective wellbeing

SWB is often conceived of as three different metrics: positive affect, negative affect, and life satisfaction (Lucas et al., 1996; Diener and Biswas-Diener, 2002; Diener et al., 2003). We had measurements of all three.

*Positive affect* (Cronbach’s *α* = 0.841, McDonald’s *ω* = 0.894) was assessed with the positive affect subscale of the Affect-Adjective Scale (Diener et al., 1985a,b). Participants rated the extent to which they experienced four positive emotions (i.e., happy, joyful, fun/enjoyment, pleased) at the moment. To mask the true focus of the study, three distractor items (interested, relaxed, excited) were included (Geenen et al., 2014). Responses were made on a scale of 1 (not at all) to 7 (extremely).

*Negative affect* (*α* = 0.886, *ω* = 0.917) was assessed with the negative affect subscale of the Affect-Adjective Scale (Diener et al., 1985a,b). Participants rated the extent to which they experienced five negative emotions (i.e., worried/anxious, angry/hostile, frustrated, depressed/blue, unhappy) at the moment. Again, two distractor items (bored, tired) were included (Geenen et al., 2014). Responses were made on a scale of 1 (not at all) to 7 (extremely).

*Life satisfaction* (*α* = 0.886, ω = 0.921) was assessed using the Satisfaction with Life Scale (Diener et al., 1985a,b). This 5-item scale asks participants to rate their agreement with statements such as “In most ways, my life is close to my ideal” and “The conditions of my life are excellent,” using a 7-point Likert scale from 1 (strongly disagree) to 7 (strongly agree).

An aggregate SWB score was calculated by standardizing and summing life satisfaction scores with positive affect scores and subtracting negative affect (Diener and Lucas, 1999).

##### 2.1.2.2 Basic psychological needs

SDT stipulates that satisfaction of three BPN—autonomy (the need to experience choice and psychological freedom), competence (the need to feel effective or a sense of mastery), and relatedness (the need to feel connected with significant others)—determines human wellbeing (Hui and Kogan, 2018). This was measured using a 9-item Basic Psychological Needs Scale (La Guardia et al., 2000; Hui and Kogan, 2018). It assessed the extent to which participants experienced each of the three BPN as being satisfied at the moment, using a 7-point scale ranging from 1 (not at all true) to 7 (very much true).

Each of the subscale consists of 3 items. *Autonomy* (*α* = 0.807, *ω* = 0.886) was assessed using items such as “I feel free to be who I am” and “I have a say in what happens.” *Competence* (*α* = 0.825, *ω* = 0.896) was assessed with items such as “I feel like a competent person” and “I feel very capable and effective.” *Relatedness* (*α* = 0.848, *ω* = 0.908) was measured with items like “I feel loved and cared about” and “I feel a lot of closeness and intimacy.” Each of the three subscales was averaged to form an index of general BPN satisfaction (*α* = 0.800, *ω* = 0.851).

##### 2.1.2.3 Blood donation intention

Blood donation intention among blood donors was measured with a single item: “How likely will you donate blood again in the future?” rated from 1 (very unlikely) to 7 (very likely).

### 2.2 Randomized trial

#### 2.2.1 Study design and participants

To examine H2-4, a single-center, parallel randomized trial was conducted. Participants were from the Donation group in the baseline survey (*n* = 601). When donors’ blood was supplied to the hospitals, the blood donation and supply system of Guangzhou Blood Center would send a standard blood utilization notification to the donors automatically, which read: “Dear Ms./Mr. XXX, your donating blood has been sent to the hospital, thank you for your donation!*”* Using the phone number provided by blood donors, the principal investigator checked the database of Guangzhou Blood Center (PAss Aladdin System, Pass Software Inc.) daily to confirm whether participants had received the above message. Once confirmed, participants were randomly assigned to two sub-groups (Intervention group or Control group) using computer-generated random numbers.

Two different online questionnaires were created, and participants were given corresponding link, which took about 4–28 days after their donation. In the second round of data collection, all participants could enter a prize draw to win a bonus packet worth CNY 3–7 Yuan (approximately US$0.5–1) as an incentive for completion of the questionnaire. Individuals who failed the attention-check item or declined to provide a phone number were excluded.

#### 2.2.2 Interventions and control conditions

The items of both the Intervention and Control questionnaires were identical to the baseline questionnaire. In the Intervention group, a gratitude reinforcement message was presented at the beginning of the questionnaire and a vignette of a patient transfusing blood was attached below; whereas questionnaire in the Control group did not present such message or vignette. The gratitude reinforcement message read as follow: “Thank you for your selfless love. Your donating blood has saved patients’ lives! Their family can be reunited and live happily after their recovery.” The links were closed 1 week after the last message being sent, based on prior experience that most blood donors would complete the questionnaire within 3 days of receiving the message and few would do so after 1 week. Blood donors would provide their cell phone number for matching if they consented.

#### 2.2.3 Follow-up and outcome measures

**Figure 1:**
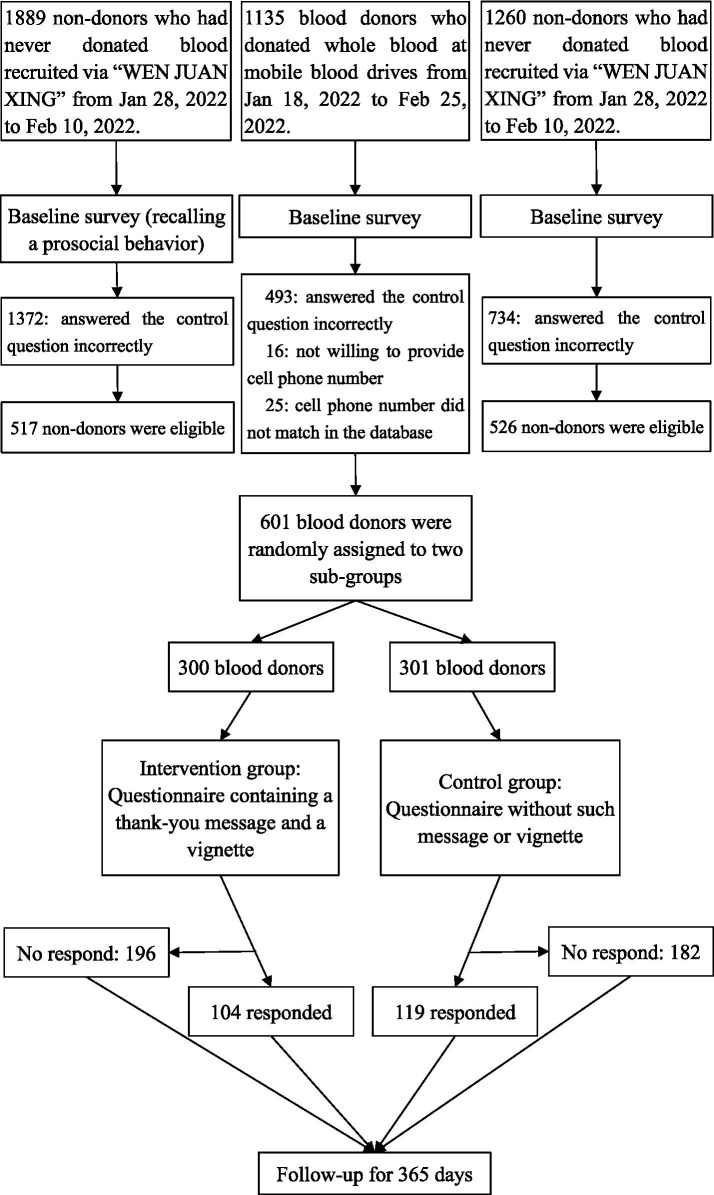
Flowchart of the study design, participant recruitment, and follow-up procedures.

SWB, BPN and blood donation intention of all participants in the Donation group were measured before (Time-1, T_1_) and after the intervention (Time-2, T_2_). Exploratory analyses conducted for descriptive purposes (e.g., effects of the gratitude reinforcement message on T_2_ subdimension outcomes, within-group comparisons over time, comparing the Intervention group at T_2_ with other groups at T_1_, examining the association between T_2_ SWB and subsequent re-donation behavior) are presented in Supplementary material 2. Figure 1 illustrates the study design.

### 2.3 Analysis

SPSS Statistics version 26 for Windows (SPSS Inc., Armonk, NY, USA) was used for all quantitative analyses. One-way analysis of variance (ANOVA), analysis of covariance (ANCOVA), multiple linear regression, and logistic regression were used. When an ANCOVA was conducted, the partial *η*^2^ value was used to estimate the effect size. When an independent-sample *t*-test was used, Cohen’s *d* value was used to estimate the effect size.

Measurement invariance of the SWB and BPN scales across donor and non-donor groups was evaluated using multi-group confirmatory factor analysis in R with the lavaan (version 0.6–20) and semTools (version 0.5–7) packages. Following established recommendations (Vandenberg and Lance, 2000), four nested models were tested sequentially: configural, metric (weak), scalar (strong), and strict invariance. Model fit was assessed using the comparative fit index (CFI) and root mean square error of approximation (RMSEA). Invariance was considered supported when changes between nested models met the following criteria: ΔCFI ≤ 0.010 and ΔRMSEA ≤ 0.015 (Cheung and Rensvold, 2002; Chen, 2007).

### 2.4 Ethical considerations

All study procedures were reviewed and approved by the Institutional Review Board of the Guangzhou Blood Center (Approval No. 202201). Digital informed consent was obtained from all participants prior to questionnaire completion. No personally identifying information was collected from non-donors during the baseline survey. For blood donors, only mobile phone numbers were collected to enable follow-up, and these were kept strictly confidential. All data were stored in a secure spreadsheet; the original dataset was saved in a password-protected folder on the principal investigator’s computer. A deidentified version of the dataset was created before any data cleaning or analysis. Variable labels and response levels were cleaned and re-coded during data preparation. The ethics committee approved both the digital consent procedure and the collection of phone numbers from donors.

This study was considered low risk, as the intervention materials (gratitude reinforcement message and vignette) were similar to communications that donors might normally encounter, aside from the randomization procedure. All data were processed anonymously to ensure confidentiality. To minimize bias, participants in the randomized trial were unaware of the study’s hypotheses and were not informed about the experimental purpose of the intervention, as such knowledge could influence questionnaire responses or re-donation behavior. Because participants necessarily viewed different questionnaire materials, the study did not involve full blinding to intervention exposure. However, blinding to the study hypotheses was maintained. The ethics committee approved this procedure. The study was conducted in accordance with the Declaration of Helsinki.

## 3 Results

### 3.1 Baseline survey

A total of 4,284 questionnaires were collected (Donation = 1,135, Recall = 1,889, and Non-recall = 1,260). After excluding invalid responses (Figure 1), 601 donors (52.9%), 517 participants in the Recall group (27.4%), and 526 in the Non-recall group (41.7%) were included in the analyses. A sensitivity analysis conducted in G*Power 3.1 indicated that the current sample size provided sufficient statistical power (1 − *β* = 0.80) to detect even small effects (*f*^2^ = 0.008) for individual regression coefficients at *α* = 0.05 (two-tailed), confirming adequate power for planned analyses (Faul et al., 2009).

Measurement invariance testing supported configural and metric invariance for both SWB and BPN scales. For the SWB scale, changes in model fit between the metric and scalar models were both within recommended thresholds. Although strict invariance was not achieved, mean comparisons remain valid. These findings indicate that the constructs were measured equivalently across groups (Supplementary Table S1).

Supplementary Table S2 presents the demographic characteristics. Donors reported an average lifetime donation frequency of 4.8 ± 5.5, and blood donation intention was high (6.42 ± 0.82). No data were missing due to the digital survey format. Age did not differ across groups (independent-sample *t*-test *p*s > 0.05).

#### 3.1.1 Factors associated with Time-1 subjective wellbeing

Supplementary Table S3 reports the means, standard deviations, and correlations for the variables. Multiple linear regression (ordinary least squares) was used to explore the association between T_1_ SWB and the variables. As shown in Table 1, after controlling for covariates, both the Donation and Recall groups were associated with higher SWB compared with the Non-recall group. T_1_ BPN satisfaction, age, income, and being married were also positively associated with T_1_ SWB.

**Table 1:** Multiple linear regression model predicting Time-1 subjective wellbeing.

Predictor	*B (SE)*	*t*	*p*	95%CI
Constant	−7.97 (0.50)		<0.001	−8.95 ~ −7.00
Group				
Donation	0.32 (0.11)	2.91	0.004	0.11 ~ 0.54
Recall	0.22 (0.01)	2.22	0.027	0.03 ~ 0.41
Non-recall	Reference			
Time-1 basic psychological needs	1.21 (0.07)	18.14	<0.001	1.08 ~ 1.34
Gender				
Male	0.01 (0.08)	0.06	0.953	−0.15 ~ 0.16
Female	Reference			
Age	0.02 (0.01)	2.81	0.005	0.01 ~ 0.03
Education level	0.06 (0.05)	1.13	0.26	−0.04 ~ 0.16
Employment status				
Employed	−0.20 (0.13)	−1.46	0.144	−0.46 ~ 0.07
Unemployed	Reference			
Income	0.24 (0.04)	6.16	<0.001	0.16 ~ 0.32
Marital status				
Married	0.57 (0.13)	4.26	<0.001	0.31 ~ 0.83
Unmarried	Reference			
Number of children	−0.02 (0.09)	−0.22	0.823	−0.19 ~ 0.15

*N* = 1,644.

Adjusted *R*^2^ = 0.24.

#### 3.1.2 Comparisons of Time-1 outcomes

A one-way ANOVA showed no significant overall differences in T_1_ SWB among the three groups (*F*_(2, 1,641)_ = 2.90, *p* = 0.056). However, Bonferroni-adjusted pairwise comparisons indicated that participants in the Recall group reported significantly higher T_1_ SWB than those in the Non-recall group (*p* = 0.024). For T_1_ BPN satisfaction, the overall ANOVA was significant (*F*_(2, 1,641)_ = 3.23, *p* = 0.040), and Bonferroni *post-hoc* tests showed that both the Donation group (*p* = 0.026) and the Recall group (*p* = 0.028) reported higher levels of T_1_ BPN satisfaction than the Non-recall group. Analyses of subdimensions further indicated significant differences in positive affect, negative affect, autonomy, and competence.

Independent-sample *t*-test results for T_1_ SWB and BPN are presented in Table 2. Participants in the Recall group reported slightly higher levels of T_1_ SWB than those in the Donation and Non-recall groups. Although some pairwise differences reached statistical significance, the corresponding effect sizes were very small (Cohen’s *ds* < 0.20). Overall, these results provide only partial support for H1.

**Table 2:** Independent-sample *t*-test results for Time-1 subjective wellbeing and basic psychological needs across groups.

Groups	*N*	Subjective wellbeing	Comparison	*p (t)*	Cohen’s *d*
		*M* ± *SD*			
Donation (1)	601	−0.05 ± 1.69	1 vs. 2	0.055 (−1.92)	0.12
Recall (2)	517	0.16 ± 1.88	2 vs. 3	0.030 (2.18)	0.14
Non-recall (3)	526	−0.10 ± 1.86	1 vs. 3	0.660 (0.44)	0.03

Groups	*N*	Basic psychological needs	Comparison	*p (t)*	Cohen’s *d*
		*M* ± *SD*			
Donation (1)	601	5.38 ± 0.54	1 vs. 2	0.952 (−0.06)	0.00
Recall (2)	517	5.38 ± 0.64	2 vs. 3	0.036 (2.10)	0.13
Non-recall (3)	526	5.30 ± 0.60	1 vs. 3	0.021 (2.32)	0.12

Supplementary Table S4 details the between-group comparisons on the subcomponents of SWB and BPN. Notably, participants in the Donation group reported higher levels of negative affect than those in both the Recall and Non-recall groups with small effect sizes.

#### 3.1.3 Mediation by basic psychological needs satisfaction at Time-1

Following the procedures identified by Baron and Kenny (1986), with sociodemographic characteristics included as control variables, a linear regression analysis showed that blood donation was positively associated with T_1_ BPN satisfaction (*B (SE)* = 0.14 (0.04), *p* = 0.001; *F*_(8,1,118)_ = 2.90, *p* = 0.003, *R*^2^ = 0.01). Regressing T_1_ SWB onto blood donation also yielded a significant effect (*B (SE)* = 0.39 (0.13), *p* = 0.003; *F*_(8,1,118)_ = 8.94, *p* < 0.001, *R*^2^ = 0.05). When both blood donation and T_1_ BPN satisfaction were entered into the model predicting SWB, BPN satisfaction remained a significant predictor (*B (SE)* = 1.17 (0.08), *p* < 0.001), whereas the direct effect of blood donation was no longer statistically significant (*B (SE)* = 0.22 (0.12), *p* = 0.073) (*F*_(9,1,117)_ = 31.11, *p* < 0.001, *R*^2^ = 0.19) (Supplementary Table S5).

Bootstrap analysis with 5,000 resamples further showed that the 95% confidence interval for the indirect effect did not include zero (95% CI [0.065, 0.278]), suggesting a significant mediating role of T_1_ BPN satisfaction (Supplementary Table S6). The mediated effect accounted for 43.6% of the total effect. Overall, these results suggest that blood donation was associated with higher T_1_ BPN satisfaction, which in turn was associated with greater T_1_ SWB. H2 is supported, as the results were consistent with a mediating role of T_1_ BPN satisfaction in the association between blood donation and T_1_ SWB.

#### 3.1.4 Factors predicting Time-1 blood donation intention

Supplementary Table S7 reports the correlations for the variables among blood donors. Supplementary Table S8 shows that blood donors’ T_1_ future donation intention was positively associated with T_1_ BPN, T_1_ SWB and donation frequency. H3 is supported.

### 3.2 Randomized trial

All 601 participants from the Donation group were successfully randomized, with 300 blood donors assigned to the Intervention group and 301 to the Control group. The average time for donated blood to be supplied to hospitals was 6.5 ± 1.8 days (range: 4–22 days). The follow-up questionnaire (T_2_) was completed on average 6.6 ± 1.9 days after donation (range: 4–15 days; time-since-donation). The overall response rate for the T_2_ questionnaire was 37.1% (223/601), with 104 respondents in the Intervention group (34.7%) and 119 in the Control group (39.5%). Retention rates did not differ significantly between groups (*χ*^2^ = 1.53, *p* = 0.217).

In addition, comparisons between responders and non-responders revealed no significant differences in baseline SWB, BPN satisfaction, or key sociodemographic characteristics (Supplementary Table S9), suggesting that attrition was unlikely to introduce substantial bias.

Baseline comparisons showed no significant differences between the Intervention and Control groups in sociodemographic characteristics, donation frequency, time-since-donation, blood donation intention, SWB, or BPN satisfaction at T_1_ (Supplementary Table S10), indicating successful randomization.

#### 3.2.1 Effects of the gratitude reinforcement message on Time-2 outcomes

The correlation results showed that T_2_ SWB was positively associated with T_2_ BPN satisfaction (0.213) and strongly associated with T_1_ SWB (0.780) (Supplementary Table S11). An ANCOVA model (Table 3) was conducted to estimate the intervention effect on T_2_ SWB while controlling for T_1_ SWB and other covariates. The intervention was not significantly associated with T_2_ SWB (*B (SE)* = 1.05 (0.66), *p* = 0.111, partial *η*^2^ = 0.01). T_2_ SWB was strongly associated with T_1_ SWB (*B (SE)* = 3.43 (0.20), *p* < 0.001, partial *η*^2^ = 0.58).

**Table 3:** ANCOVA model predicting Time-2 subjective wellbeing.

Predictor	*B (SE)*	*t*	*p*	95%CI	Partial *η*^2^
Intercept	3.23 (2.30)	1.41	0.161	−1.30 ~ 7.77	0.01
Group					
Intervention	1.05 (0.66)	1.60	0.111	−0.24 ~ 2.34	0.01
Control	Reference				
Control variables					
Time-1 subjective wellbeing	3.43 (0.20)	17.23	<0.001	3.03 ~ 3.82	0.58
Donation frequency	−0.09 (0.06)	−1.57	0.118	−0.21 ~ 0.02	0.01
Gender	−1.16 (0.68)	−1.71	0.088	−2.5 ~ 0.17	0.01
Age	0.09 (0.06)	1.60	0.111	−0.02 ~ 0.20	0.01
Education level	−0.27 (0.38)	−0.73	0.467	−1.02 ~ 0.47	0.00
Employment status	−1.35 (0.98)	−1.37	0.171	−3.28 ~ 0.59	0.01
Income	−0.70 (0.29)	−2.41	0.017	−1.27 ~ −0.13	0.03
Marital status	2.33 (1.11)	2.09	0.037	0.14 ~ 4.53	0.02
Number of children	−0.39 (0.72)	−0.54	0.590	−1.82 ~ 1.04	0.00
Time-since-donation	−0.02 (0.18)	−0.09	0.928	−0.37 ~ 0.34	0.00

*N* = 223.

Adjusted *R*^2^ = 0.63.

Another ANCOVA model (Table 4) predicting T_2_ BPN satisfaction showed a significant intervention effect (*B (SE)* = 0.18 (0.05), *p* = 0.001). Although statistically significant, the effect size was small-to-moderate (partial *η*^2^ = 0.05). T_2_ BPN satisfaction was strongly associated with T_1_ BPN satisfaction (*B (SE) =* 0.68 (0.06), *p* < 0.001, partial *η*^2^ = 0.42). H4a is partially supported, as the intervention significantly increased T_2_ BPN satisfaction but did not produce a statistically significant improvement in T_2_ SWB.

**Table 4:** ANCOVA model predicting Time-2 basic psychological needs satisfaction.

Predictor	*B (SE)*	*t*	*p*	95%CI	Partial *η*^2^
Intercept	1.61 (0.37)	4.32	<0.001	0.88 ~ 2.35	0.08
Group					
Intervention	0.18 (0.05)	3.24	0.001	0.07 ~ 0.29	0.05
Control	Reference				
Control variables					
Time-1 basic psychological needs	0.68 (0.06)	12.29	<0.001	0.57 ~ 0.79	0.42
Donation frequency	0.00 (0.01)	−0.85	0.394	−0.01 ~ 0.01	0.00
Gender	−0.01 (0.06)	−0.19	0.852	−0.12 ~ 0.10	0.00
Age	0.00 (0.00)	0.49	0.623	−0.01 ~ 0.01	0.00
Education level	0.02 (0.03)	0.76	0.445	−0.04 ~ 0.09	0.00
Employment status	0.01 (0.08)	0.10	0.921	−0.15 ~ 0.16	0.00
Income	0.00 (0.02)	0.12	0.908	−0.05 ~ 0.05	0.00
Marital status	−0.13 (0.09)	−1.39	0.166	−0.32 ~ 0.05	0.01
Number of children	0.03 (0.06)	0.47	0.641	−0.09 ~ 0.15	0.00
Time-since-donation	0.00 (0.01)	−0.27	0.785	−0.03 ~ 0.03	0.00

*N* = 223.

Adjusted *R*^2^ = 0.43.

Exploratory ANCOVA analyses of SWB and BPN subdimensions are reported in Supplementary material 2. Briefly, the intervention showed no significant effects on T_2_ positive affect, negative affect, or life satisfaction, but small effects were observed for autonomy and competence, while all T_2_ subdimensions were strongly associated with their corresponding T_1_ levels.

#### 3.2.2 Mediating role of basic psychological needs satisfaction at Time-2

Supplementary Table S12 presents the linear regression models examining the associations among group assignment, T_2_ BPN satisfaction, and T_2_ SWB. These models were estimated after controlling for baseline levels of SWB and BPN satisfaction at T_1_, time-since-donation, and other prespecified covariates. The intervention significantly predicted T_2_ BPN satisfaction (*B (SE)* = 0.26 (0.09), *p* = 0.005), and T_2_ BPN satisfaction was positively associated with T_2_ SWB (*B (SE)* = 1.27 (0.49), *p* = 0.010). The bootstrapped 95% confidence interval for the indirect effect did not include zero (95%CI [0.043, 0.901]), suggesting evidence for an indirect pathway between the intervention and T_2_ SWB through T_2_ BPN satisfaction (Supplementary Table S13). The indirect effect accounted for 36.4% of the total effect.

Additional analyses (Supplementary Tables S14, S15) indicated that competence satisfaction also showed evidence of an indirect pathway, whereas autonomy and relatedness did not (data not shown). The mediated effect of competence accounted for 32.2% of the total effect. H4b is partially supported, as evidence for an indirect pathway through T_2_ BPN satisfaction was observed, while the total effect of the intervention on T_2_ SWB remained small and did not reach statistical significance.

## 4 Discussion

The present research should be viewed as a preliminary step in understanding the relationship between blood donation and SWB. Our findings align with previous research, showing that both performing and recalling prosocial behavior are associated with higher SWB (Ko et al., 2021). In the baseline analyses, T_1_ BPN satisfaction statistically mediated the association between blood donation and T_1_ SWB, which is congruent with previous studies reporting that BPN plays a mediating role in the relationship between prosocial behavior and wellbeing (Dunn et al., 2014; Geenen et al., 2014; Hui and Kogan, 2018). These results suggest that blood donation may be linked to higher SWB when the BPN of autonomy, competence, and relatedness are satisfied. These findings have potential implications for policymakers. In Mainland China, the gap between blood supply and demand has become increasingly prominent. One possible reason for low blood donor recruitment may be limited public awareness of the potential psychological benefits associated with blood donation. When donors experience satisfaction of their needs for autonomy, competence, and relatedness, they may be more likely to perceive emotional rewards from blood donation. Blood centers may therefore consider incorporating communication strategies that highlight these psychological benefits, potentially increasing the likelihood that one or more of these needs is fulfilled during the donation experience.

Blood donors reported lower T_1_ SWB than non-donors in the Recall group, although this difference was not statistically significant. Additionally, blood donors experienced significantly higher negative affect than non-donors in both the Recall and Non-recall groups. One possible explanation is that blood donors completed the baseline questionnaire immediately after donating blood. Pain associated with the finger-stick blood test and needle insertion, fear before and/or during donation, post-donation discomfort (including possible vasovagal reactions), or anxiety about unknown blood test results may have contributed to the higher negative affect. However, unlike positive emotions, which may last for days or weeks, negative emotions often dissipate within minutes or hours (Hinrichs et al., 2008). By the time donors completed the second questionnaire, which was sent 4–22 days after donation, negative affect may have subsided and positive affect may have increased. Correspondingly, higher SWB levels were observed at T_2_ within the Intervention group, although between-group differences in SWB were not statistically significant.

Results from the randomized trial showed that T_2_ SWB was strongly associated with T_1_ SWB, indicating substantial short-term stability in SWB, which is in line with previous studies (Falk and Graeber, 2020; Xiong et al., 2023). After controlling for baseline levels and other covariates, the intervention did not show a statistically significant direct effect on T_2_ SWB. However, the intervention was positively associated with T_2_ BPN satisfaction, and BPN satisfaction was positively associated with T_2_ SWB, suggesting a potential indirect pathway linking the intervention to wellbeing. Further analyses indicated that this pathway was primarily driven by competence satisfaction. This finding suggests that the gratitude reinforcement message may have mainly operated by strengthening donors’ sense of competence—reminding them that their donation had a meaningful and potentially life-saving impact. Within the framework of SDT, feeling effective in helping others may represent an important psychological reward associated with blood donation. However, because the total effect of the intervention on T_2_ SWB was not statistically significant, these mediation findings should be interpreted cautiously. The results therefore suggest a possible indirect pathway through competence satisfaction rather than providing definitive evidence of a causal intervention effect on wellbeing.

At the same time, the relatively modest effects observed in this study may reflect the limited intensity of the intervention, which consisted only of a brief written message and vignette. This type of feedback is commonly used in blood donation services because it is low-cost and easily scalable. For example, some blood services send personalized SMS notifications informing donors when their blood has been used for a patient, a feedback mechanism shown to strengthen donors’ sense of competence and increase return donation rates (Gemelli et al., 2017; Pongsananurak et al., 2020; Shehu et al., 2024). However, although such approaches are practical and widely implemented, they may not be sufficiently powerful to produce substantial improvements in SWB. Future interventions may therefore benefit from incorporating more immersive or emotionally engaging forms of feedback, such as short video messages from patients or visual storytelling about how donated blood saves lives.

At T_1_, both SWB and BPN satisfaction were positively associated with donors’ intention to donate again, consistent with theories proposing a positive feedback loop between prosocial behavior and wellbeing (Thoits and Hewitt, 2001; Aknin et al., 2012; Brethel-Haurwitz and Marsh, 2014). This finding suggests that donors who experience greater psychological fulfillment may be more motivated to consider returning. However, translating intention into actual donation behavior may be particularly complex in the context of blood donation. Blood donation represents a distinct type of formal prosocial behavior (Borgonovi, 2008). It is embedded within a unique socio-cultural context where traditional beliefs may frame it as potentially depleting (Ou-Yang et al., 2017; Ou-Yang et al., 2020a,b), and it involves a specific set of tangible costs and procedural steps that differentiate it from both monetary giving and extraordinary acts like anonymous organ donation (Rhoads et al., 2021). Moreover, donors’ motivations are multifaceted, extending beyond pure altruism to include warm-glow and other factors (Bednall and Bove, 2011; Ferguson et al., 2012; Ferguson, 2015), which may decouple the immediate emotional reward from the long-term behavioral loop. Consistent with this complexity, our exploratory analyses showed that past donation frequency, a marker of established habit and donor identity (Ferguson and Lawrence, 2019), was a strong predictor of future re-donation. In contrast, although the initial association between SWB and intention was observed, analyses of actual re-donation behavior did not reveal a consistent or theoretically clear pattern. Therefore, the associations involving re-donation should be interpreted cautiously, as they likely reflect complex, unmeasured contextual factors rather than a straightforward breakdown of the prosociality-SWB loop. Future longitudinal research is needed to examine how psychological fulfillment, habitual donation, and contextual factors jointly shape sustained blood donation behavior.

### 4.1 Limitations

First, the initial registration numbers were high, but only 37% of donors completed the second questionnaire. Although attrition did not appear to alter the main findings and retention rates were similar between groups, the relatively low follow-up rate may have reduced statistical power for detecting between-group differences and may limit generalizability of the results to donors who complete follow-up assessments. Second, donor and non-donor data were collected from different sources. Although scalar invariance was established for both SWB and BPN scales, the lack of strict invariance suggests differences in measurement error across groups, which may affect the precision of cross-group comparisons. Third, blood donors represent a generally healthy population because eligibility requires meeting specific health criteria. Thus, observed SWB differences between donors and non-donors may partly reflect baseline health disparities rather than donation effects alone (Kushlev et al., 2020). Future studies incorporating health-matched control groups would help disentangle these effects. Fourth, although the randomized design strengthens causal inference, the intervention was relatively mild and may have been insufficient to produce detectable changes in SWB over the short follow-up period. Future studies using stronger interventions may help clarify the causal effects of psychological feedback on donor wellbeing. Fifth, the generalizability of our findings is constrained by the socio-historical and geographical context of data collection. The study was conducted in early 2022, during the latter phase of the COVID-19 pandemic in China, when heightened health concerns, mobility restrictions, and changes in donation logistics may have influenced both SWB and helping-related motivations. Furthermore, all participants were recruited from Guangzhou, and donor and non-donor samples were obtained through different recruitment channels (mobile blood drives vs. online survey platforms). These contextual factors may limit the extent to which the present results can be generalized to other regions, post-pandemic conditions, or populations with different donation practices. Finally, this study focused on donors at mobile drives. In Mainland China, group-organized donations (where organizations arrange for employees or members to donate collectively) are common (Ou-Yang et al., 2020a,b). Given that China is a collectivist society, group blood donors may have different perspectives on SWB and BPN satisfaction. Future research could explore the effects of group dynamics on SWB and donation behavior.

## 5 Conclusion

In conclusion, this study provides preliminary evidence that blood donation, a formal prosocial behavior, is associated with SWB. This finding suggests that satisfaction of BPN, particularly competence may represent one psychological pathway linking blood donation to SWB. These insights may help inform strategies for blood donor recruitment and retention, which are essential for maintaining a stable blood supply. Future research using stronger interventions and longer follow-up periods is needed to further clarify the causal relationship between blood donation and SWB.

## Data Availability

The raw data supporting the conclusions of this article will be made available by the authors, without undue reservation.

## Footnotes

1
https://www.wjx.cn/
