# Supplementary material for: Blood donation and subjective wellbeing: a cross-sectional survey and a randomized trial
Source: Front Psychol. 2026 Mar 27;17:1795243. doi: 10.3389/fpsyg.2026.1795243 (PMC13065722; doi:10.3389/fpsyg.2026.1795243)
Supplement: Supplementary file 2 [file Data_Sheet_2.pdf]

S.1 Measurement invariance testing across donor and non-donor groups

	Model	CFI	RMSEA	ΔCFI	ΔRMSEA	Conclusion
Subjective well-being	Configural	.945	.079	-	-	Supported
	Metric (weak)	.940	.078	-.005	.001	Supported
	Scalar (strong)	.936	.080	-.004	.002	Supported
	Strict	.909	.090	-.027	.010	Not supported
Basic psychological needs satisfaction	Configural	.989	.051	-	-	Supported
	Metric (weak)	.986	.052	-.003	.001	Supported
	Scalar (strong)	.983	.053	-.003	.001	Supported
	Strict	.933	.098	-.050	.045	Not supported

N = 1644

S.2 Basic characteristics of all participants in baseline survey

	Donation	Recall	Non-recall
Sample sizes	601	517	526
Gender			
Female	258 (42.9)	279 (54.0)	339 (64.4)
Male	343 (57.1)	238 (46.0)	187 (35.6)
Age	30.8 ± 9.5	30.1 ± 6.0	30.0 ± 7.0
Education level [a]			
Junior high or below	44 (7.3)	8 (1.5)	2 (.4)
Secondary school	115 (19.1)	23 (4.4)	17 (3.2)
Junior college	176 (29.3)	46 (8.9)	51 (9.7)
College	234 (38.9)	404 (78.1)	415 (78.9)
Master or above	32 (5.3)	36 (7.0)	41 (7.8)
Employment status [b]			
Employed	476 (79.2)	465 (89.9)	490 (93.2)
Unemployed	125 (20.8)	52 (10.1)	36 (6.8)
Household per capita monthly income [a]			
Less than CNY 3000 (US$418)	77 (12.8)	26 (5.0)	15 (2.9)
CNY 3000-7999 (US$418-1113)	128 (21.3)	80 (15.5)	57 (10.8)
CNY 8000-14999 (US$1113-2088)	194 (32.3)	152 (29.4)	159 (30.2)
CNY 15000-29999 (US$2088-4177)	135 (22.5)	186 (26.0)	211 (40.1)
More than CNY 30000 (US$4177)	67 (11.1)	73 (14.1)	84 (16.0)
Marital status [c]			
Married	212 (35.3)	373 (72.1)	406 (77.2)
Unmarried	389 (64.7)	144 (27.9)	120 (22.8)
Number of Children [a]			
None	395 (65.7)	175 (33.8)	146 (27.8)
1	126 (21.0)	299 (57.8)	309 (58.7)
2	67 (11.1)	41 (7.9)	66 (12.5)
3 or more	13 (2.2)	2 (.4)	5 (1.0)

N = 1644

a Ordinal categorical variable

b Dichotomously coded: Unemployed = 0 (unemployed, retiree, and student), Employed = 1 (employed full-time or part-time job, and freelance)

c Dichotomously coded: Unmarried = 0 (single/never been married, separated, divorced, or widowed), Marred = 1 (married or in a domestic partnership)

S.3 Means, standard deviations, and correlations for the variables in baseline survey

	Mean (SD)	1	2	3	4	5	6	7	8	9	10	11
1.Subjective well-being	.00 (1.81)	1										
2.Basic psychological needs	5.35 (.59)	.42**	1									
3.Autonomy	5.95 (.72)	.26**	.71**	1								
4.Competence	4.44 (.87)	.22**	.73**	.33**	1							
5.Relatedness	5.67 (.89)	.42**	.73**	.30**	.22**	1						
6.Gender	-	-.01	.02	.03	.00	.01	1					
7.Age	30.3 (7.7)	.13**	-.03	.01	-.03	-.04	-.17**	1				
8.Education level	-	.07**	.03	.00	-.03	.10**	.14**	-.18**	1			
9.Employment status	-	-.08**	-.03	-.01	-.01	-.05*	.03	-.39**	.04	1		
10.Income	-	.22**	.09**	.10**	.00	.09**	.02	.11**	.33**	-.19**	1	
11.Marital status	-	-.22**	-.07**	-.04	-.01	-.10**	-.04	-.41**	-.13**	.43**	-.31**	1
12.Number of Children	-	.18**	.07**	.07**	.02	.08**	.01	.51**	-.03	-.33**	.21**	-.73**

N = 1644

* p<.05; ** p<.01

S.4 Independent-sample t-test results of other factors across groups

Groups	Positive affect	Comparison	*p (t)*	*Cohen's d*	Negative affect	Comparison	*p (t)*	*Cohen's d*
	M±SD				*M±SD*			
Donation (1)	-.03±.83	1 *vs.* 2	.538 (-.62)	.04	.11±.83	1 *vs.* 2	<.001 (4.1)	.24
Recall (2)	.06±.79	2 *vs.* 3	.004 (2.86)	.18	-.09±.82	2 *vs.* 3	.435 (-.78)	.05
Non-recall (3)	-.09±.85	1 *vs.* 3	.022 (2.30)	.14	-.05±.84	1 *vs.* 3	.001 (3.22)	.20

Groups	Life satisfaction	Comparison	*p (t)*	*Cohen's d*	Autonomy	Comparison	*p (t)*	*Cohen's d*
	M±SD				*M±SD*			
Donation (1)	.04±.79	1 *vs.* 2	.634 (.48)	.03	6.08±.66	1 *vs.* 2	<.001 (4.25)	.26
Recall (2)	.01±.89	2 *vs.* 3	.209 (1.26)	.08	5.90±.76	2 *vs.* 3	.411 (.82)	.05
Non-recall (3)	-.05±.83	1 *vs.* 3	.060 (1.88)	.11	5.86±.72	1 *vs.* 3	<.001 (5.32)	.32

Groups	Competence	Comparison	*p (t)*	*Cohen's d*	Relatedness	Comparison	*p (t)*	*Cohen's d*
	M±SD				*M±SD*			
Donation (1)	5.46±.98	1 *vs.* 2	.062 (-1.87)	.11	4.60±.76	1 *vs.* 2	.106 (-1.62)	.10
Recall (2)	5.55±.75	2 *vs.* 3	<.001 (4.73)	.29	4.69±1.05	2 *vs.* 3	.656 (-.45)	.03
Non-recall (3)	5.32±.83	1 *vs.* 3	.015 (2.44)	.15	4.72±.84	1 *vs.* 3	.017 (-2.34)	.14

$N_{(Donation)} = 601$; $N_{(Recall)} = 517$; $N_{(Non\text{-}recall)} = 526$

S.5 Linear regression models examining blood donation, Time-1 basic psychological needs satisfaction, and Time-1 subjective well-being in baseline survey

Independent variables	Dependent variables								
	Time-1 Basic psychological needs			Time-1 Subjective well-being			Time-1 Subjective well-being		
	B (SE)	*t*	*p*	B (SE)	*t*	*p*	B (SE)	*t*	*p*
Constant	5.21 (.12)	42.58	<.001	-1.90 (.37)	-5.12	<.001	-8.01 (.56)	-14.44	<.001
Donation group	.14 (.04)	3.32	.001	.39 (.13)	2.95	.003	.22 (.12)	1.80	.073
Time-1 Basic psychological needs	-	-	-	-	-	-	1.17 (.08)	14.00	<.001
Control variables									
Gender	-.04 (.04)	1.10	.271	.10 (.11)	.91	.364	.05 (.10)	.52	.601
Age	-.01 (.00)	-1.69	.091	.02 (.01)	1.95	.051	.02 (.01)	2.82	.005
Education level	.01 (.02)	.25	.799	.09 (.07)	1.32	.186	.08 (.06)	1.33	.185
Employment status	-.02 (.06)	-.34	.737	-.21 (.17)	-1.21	.226	-.19 (.16)	-1.17	.242
Income	.01 (.02)	.36	.721	.17 (.05)	3.39	.001	.17 (.05)	3.52	<.001
Marital status	.10 (.10)	1.71	.088	.52 (.18)	2.94	.003	.40 (.16)	2.47	.014
Number of Children	.07 (.04)	1.80	.072	.06 (.11)	.55	.581	-.02 (.10)	-.16	.876
Adjusted R^2	.01			.05			.19		
F	$F_{(8,1118)} = 2.90$, $p = .003$			$F_{(8,1118)} = 8.94$, $p < .001$			$F_{(9,1117)} = 31.11$, $p < .001$		

N = 1127

S.6 Mediation estimates of blood donation on Time-1 subjective well-being through Time-1 basic psychological needs satisfaction

Path	Effect	Estimate (SE)	95% (CI)	*t/z*	*p*
Blood donation → Time-1 BPN satisfaction → Time-1 SWB	Indirect (a × b)	.169 (.055)	.065 ~ .278	3.092	.002
Blood donation → Time-1 BPN satisfaction	X → M (a)	.144 (.043)	.059 ~.229	3.323	.001
Time-1 BPN satisfaction → Time-1 SWB	M → Y (b)	1.173 (.084)	1.009 ~1.338	14.002	<.001
Blood donation → Time-1 SWB	Direct (c')	.219 (.122)	-.020 ~ .458	1.798	.073
Blood donation → Time-1 SWB	Total (c)	.388 (.131)	.130 ~.646	2.951	.003

BPN, Basic psychological needs; SWB, Subjective well-being

Bootstrap confidence intervals were estimated using 5,000 resamples.

S.7 Correlations for the variables among blood donors in baseline survey

	1	2	3	4	5	6	7	8	9	10	11	12	13
1.Blood donation intention	1												
2.Subjective well-being	.28**	1											
3.Basic psychological needs	.43**	.29**	1										
4.Autonomy	.36**	.19**	.59**	1									
5.Competence	.37**	.16**	.74**	.13**	1								
6.Relatedness	.14**	.25**	.67**	.23**	.18**	1							
7.Donation frequency	.11**	.10*	-.08	-.01	-.07	-.06	1						
8.Gender	-.04	.02	-.00	.04	-.01	-.02	-.07	1					
9.Age	.13**	.19**	.06	.12**	.00	.01	.45**	-.16**	1				
10.Education level	-.10*	-.01	-.10*	-.01	-.13**	-.04	.01	.08	-.17**	1			
11.Employment status	-.04	.01	-.01	-.03	-.02	.04	-.18**	.19**	-.47**	.28**	1		
12.Income	-.04	.08	-.08*	.08	-.10*	-.10*	.09*	-.08	.14**	.26**	-.11**	1	
13.Marital status	-.14**	-.16**	-.10*	-.18**	-.03	-.02	-.21**	.12**	-.62**	.14**	.33**	-.20**	1
14.Number of Children	.15**	.17**	.11**	.18**	.03	.03	.18**	-.08	.62**	-.26**	-.27**	.10*	-.75**

N = 1644

* p<.05; ** p<.01

S.8 Multiple linear regression analysis of factors associated with Time-1 blood donation intention among blood donors

Predictor	*B (SE)*	*t*	*p*	*95%CI*
Constant	3.22 (.37)	8.95	<.001	2.60~4.05
Subjective well-being	.07 (.02)	3.78	<.001	.03~.11
Basic psychological needs	.59 (.06)	10.12	<.001	.47~.70
Donation frequency	.02 (.01)	2.93	.004	.01~.03
Gender				
Male	.06 (.06)	.93	.355	-.06~.18
Female	reference			
Age	.00 (.01)	-.35	.728	-.01~.01
Education level	-.04 (.03)	-1.14	.256	-.10~.03
Employment status				
Employed	-.04 (.09)	-.41	.683	-.21~.14
Unemployed	reference			
Income	-.02 (.03)	-.68	.499	-.07~.04
Marital status				
Married	.06 (.10)	.63	.529	-.13~.26
Unmarried	reference			
Number of Children	.04 (.06)	.59	.557	-.09~.16

N = 601

Adjusted $R^2 = .23$

S.9 Baseline characteristics of responders and non-responders in randomized trial

	Responders	Non-responders	*p*
Sample sizes	223 (37.1)	378 (62.9)	
Time-1 Subjective well-being	.0 ± 1.7	-.1 ± 1.7	$p_{(t)}$ = .449 (.76) [a]
Time-1 Basic psychological needs satisfaction	5.4 ± .5	5.4 ± .6	$p_{(t)}$ = .950 (.06) [a]
Time-1 Blood donation intention	6.4 ± .9	6.5 ± .8	$p_{(t)}$ = .273 (-1.09) [a]
Gender			$p_{(\chi 2)}$ = .369 (.81) [b]
Female	101 (45.3)	157 (41.5)	
Male	122 (54.7)	221 (58.5)	
Age	31.7 ± 10.2	30.2 ± 9.0	$p_{(t)}$ = .078 (1.77) [a]
Education level [c]			$p_{(\chi 2)}$ = .143 (6.88) [a]
Junior high or below	10 (4.5)	34 (9.0)	
Secondary school	43 (19.3)	72 (19.0)	
Junior college	60 (26.9)	116 (30.7)	
College	98 (43.9)	136 (36.0)	
Master or above	12 (5.4)	20 (5.3)	
Employment status [d]			$p_{(\chi 2)}$ = .113 (2.51) [b]
Employed	169 (75.8)	307 (81.2)	
Unemployed	54 (24.2)	71 (18.8)	
Household per capita monthly income [c]			$p_{(\chi 2)}$ = .146 (6.81) [b]
Less than CNY 3000 (US$418)	34 (15.2)	43 (11.4)	
CNY 3000-7999 (US$418-1113)	47 (21.1)	81 (21.4)	
CNY 8000-14999 (US$1113-2088)	65 (29.1)	129 (34.1)	
CNY 15000-29999 (US$2088-4177)	45 (20.2)	90 (23.8)	
More than CNY 30000 (US$4177)	32 (14.3)	35 (9.3)	
Marital status [e]			$p_{(\chi 2)}$ = .443 (.59) [b]
Married	83 (37.2)	129 (34.1)	
Unmarried	140 (62.8)	249 (65.9)	
Number of Children [c]			$p_{(\chi 2)}$ = .373 (3.12) [b]
None	138 (61.9)	257 (68.0)	
1	55 (24.7)	71 (18.8)	
2	25 (11.2)	42 (11.1)	
3 or more	5 (2.2)	8 (2.1)	
Donation frequency	5.2 ± 5.5	4.6 ± 5.4	$p_{(t)}$ = .232 (1.32) [a]

N = 601

a Independent-sample *t*-test *p*

b Chi-square test *p*

c Ordinal categorical variable

d Dichotomously coded: Unemployed = 0 (unemployed, retiree, and student), Employed = 1 (employed full-time or part-time job, and freelance)

e Dichotomously coded: Unmarried = 0 (single/never been married, separated, divorced, or widowed), Marred = 1 (married or in a domestic partnership)

S.10 Basic characteristics of respondents in randomized trial

	Intervention	Control	p
Sample sizes	104	119	
Time-1 Subjective well-being	.08 ± 1.7	-.03 ± 1.7	$p_{(t)}$ = .644 (.46) [a]
Time-1 Basic psychological needs satisfaction	5.4 ± .5	5.4 ± .5	$p_{(t)}$ = .889 (-.14) [a]
Time-1 Blood donation intention	6.4 ± .9	6.4 ± .9	$p_{(t)}$ = .979 (-.03) [a]
Gender			$p_{(\chi2)}$ = .187 (1.74) [b]
Female	52 (42.6)	49 (42.2)	
Male	52 (42.6)	70 (58.8)	
Age	31.7 ± 10.6	31.6 ± 9.9	$p_{(t)}$ = .986 (.02) [a]
Education level [c]			$p_{(\chi2)}$ = .593 (2.80) [b]
Junior high or below	3 (2.9)	7 (5.9)	
Secondary school	17 (16.3)	26 (21.8)	
Junior college	28 (26.9)	32 (26.9)	
College	50 (48.1)	48 (40.3)	
Master or above	6 (5.8)	6 (5.0)	
Employment status [d]			$p_{(\chi2)}$ = .377 (.78) [b]
Employed	76 (73.1)	93 (78.2)	
Unemployed	28 (26.9)	26 (21.8)	
Household per capita monthly income [c]			$p_{(\chi2)}$ = .261 (5.27) [b]
Less than CNY 3000 (US$418)	16 (13.4)	18 (17.3)	
CNY 3000-7999 (US$418-1113)	30 (25.2)	17 (16.3)	
CNY 8000-14999 (US$1113-2088)	38 (31.9)	27 (26.0)	
CNY 15000-29999 (US$2088-4177)	20 (16.8)	25 (24.0)	
More than CNY 30000 (US$4177)	15 (12.6)	17 (16.3)	
Marital status [e]			$p_{(\chi2)}$ = .844 (.04) [b]
Married	38 (36.5)	45 (37.8)	
Unmarried	66 (63.5)	74 (62.2)	
Number of Children [c]			$p_{(\chi2)}$ = .214 (4.48) [b]
None	66 (63.5)	72 (60.5)	
One	26 (25.0)	29 (24.4)	
Two	8 (7.7)	17 (14.3)	
Three or more	4 (3.8)	1 (.8)	
Donation frequency	6.4 ± 7.0	5.4 ± 5.9	$p_{(t)}$ = .229 (1.21) [a]
Time-since-donation	6.5 ± 1.7	6.7 ± 2.0	$p_{(t)}$ = .285 (-1.07) [a]

N = 223

a Independent-sample *t*-test *p*

b Chi-square test *p*

c Ordinal categorical variable

d Dichotomously coded: Unemployed = 0 (unemployed, retiree, and student), Employed = 1 (employed full-time or part-time job, and freelance)

e Dichotomously coded: Unmarried = 0 (single/never been married, separated, divorced, or widowed), Marred = 1 (married or in a domestic partnership)

S.11 means, standard deviations, and correlations for the variables in randomized trial

	Mean (SD)	1	2	3	4	5	6	7	8	9	10	11	12	13	14	15	16	17	18	19	20	21
1.T_2 Subjective well-being	1.55 (7.88)	1																				
2.T_2 Basic psychological needs	5.46 (.53)	.21**	1																			
3.T_2 Autonomy	6.06 (.74)	.22**	.67**	1																		
4.T_2 Competence	5.68 (.79)	.20**	.72**	.32**	1																	
5.T_2 Relatedness	4.65 (.84)	.03	.63**	.09	.13	1																
6.T_1 Subjective well-being	.02 (1.72)	.78**	.14*	.16*	.14*	-.01	1															
7.T_1 Basic psychological needs	5.38 (.51)	.22**	.65**	.49**	.50**	.32**	.24**	1														
8.T_1 Autonomy	6.08 (.70)	.16*	.44**	.60**	.20**	.11	.09	.61**	1													
9.T_1 Competence	5.51 (1.00)	.07	.39**	.14*	.52**	.13	.15*	.72**	.08	1												
10.T_1 Relatedness	4.56 (.66)	.24**	.43**	.28**	.15*	.44**	.23**	.57**	.22**	.06	1											
11.T_1 Donation intention	6.38 (.90)	.24**	.38**	.25**	.42**	.10	.26**	.51**	.34**	.45**	.14*	1										
12. T_2 Donation intention	6.46 (.74)	.19**	.19**	.21**	.17**	.00	.19**	.30**	.36**	.16*	.08	.35**	1									
13. Donation frequency	5.8 (6.4)	-.01	-.12	-.07	-.08	-.10	.02	-.13	-.05	-.09	-.11	.06	.13	1								
14.Gender	-	.10	.03	-.05	.07	.03	.06	-.02	.01	.05	-.13	-.01	.05	.00	1							
15.Age	31.7 (10.2)	.17*	.00	.11	.07	-.15*	.15*	.05	.16*	.00	-.05	.13	.16*	.49**	-.16*	1						
16.Education level	-	-.02	.00	.06	-.02	-.04	.06	-.09	-.01	-.13*	.01	-.09	-.08	.03	.12	-.13	1					
17.Employment status	-	.12	.06	.04	.00	.08	.14*	.04	-.00	-.00	.09	-.04	-.03	-.26**	.20**	-.53**	.25**	1				
18.Income	-	.00	-.06	.04	-.04	-.12	.11	-.11	.03	-.14*	-.08	-.09	-.03	.18**	-.05	.26**	.31**	-.15*	1			
19.Marital status	-	-.20**	.00	-.05	-.02	.07	-.14*	-.11	-.21**	.00	-.04	-.13*	-.17*	-.27**	.20**	-.62**	.10	.39**	-.27**	1		
20.Number of Children	-	.18**	.01	.03	.03	-.03	.13	.08	.16*	-.00	.02	.10	.15*	.18**	-.14*	.65**	-.22**	-.35**	.15*	-.77**	1	
21.Donation behavior	-	-.03	.03	.01	.01	.04	-.01	-.00	-.01	-.01	.01	.00	.01	.27**	-.05	.08*	.02	-.10*	.06	-.03	.01	1
22.Time-since-donation	6.6 (1.9)	-.02	-.10	-.10	-.08	-.02	.000	-.11	-.12	-.05	-.04	.08	-.15*	.04	.06	-.11	.06	-.13*	-.05	-.09	-.12	-.08

N = 223

* p<.05; ** p<.01

S.12 Linear regression models examining intervention group, Time-2 basic psychological needs satisfaction, and Time-2 subjective well-being in randomized trial

Independent variables	Dependent variables								
	Time-2 Basic psychological needs			Time-2 Subjective well-being			Time-2 Subjective well-being		
	B (SE)	*t*	*p*	B (SE)	*t*	*p*	B (SE)	*t*	*p*
Constant	1.57 (.38)	4.11	<.001	3.34 (4.59)	.73	.467	-.04 (4.70)	-.01	.993
Intervention group	.18 (.06)	3.23	.001	1.05 (.66)	1.60	.112	.67 (.66)	1.01	.316
Time-2 basic psychological needs	-	-	-	-	-	-	2.15 (.82)	2.63	.009
Control variables									
Time-1 subjective well-being	-.01 (.02)	-.45	.657	3.43 (.20)	16.78	<.001	3.44 (.20)	17.08	<.001
Time-1 basic psychological needs	.69 (.06)	12.06	<.001	-.02 (.68)	-.03	.979	-1.49 (.88)	-1.71	.09
Donation frequency	.00 (.01)	-.87	.385	-.10 (.06)	-1.55	.122	-.09 (.06)	-1.41	.159
Gender	-.01 (.06)	-.22	.83	-1.16 (.68)	-1.71	.089	-1.14 (.67)	-1.7	.092
Age	.00 (.01)	.56	.577	.09 (.06)	1.59	.113	.08 (.06)	1.51	.132
Education level	.02 (.03)	.76	.447	-.28 (.38)	-.73	.468	-.33 (.37)	-.88	.383
Employment status	.00 (.08)	-.01	.99	-1.35 (.98)	-1.37	.172	-1.35 (.97)	-1.39	.167
Income	.00 (.03)	.16	.874	-.70 (.29)	-2.38	.018	-.71 (.29)	-2.44	.015
Marital status	-.13 (.09)	-1.37	.174	2.34 (1.13)	2.08	.039	2.61 (1.12)	2.34	.020
Number of Children	.03 (.06)	.46	.643	-.39 (.73)	-.54	.591	-.45 (.72)	-.63	.529
Time-since-donation	.00 (.02)	-.26	.797	-.02 (.18)	-.09	.927	-.01 (.18)	-.05	.963
Adjusted R^2	.43			.63			.64		
F	$F_{(12,210)} = 14.74$, $p < .001$			$F_{(12,210)} = 32.10$, $p < .001$			$F_{(13,209)} = 31.00$, $p < .001$		

N = 223

S.13 Mediation estimates of intervention on Time-2 subjective well-being through Time-2 basic psychological needs satisfaction

Path	Effect	Estimate (SE)	95% (CI)	*t/z*	*p*
Intervention → Time-2 BPN satisfaction → Time-2 SWB	Indirect (a × b)	.381 (.225)	.043 ~ .901	1.697	.090
Intervention →Time-2 BPN satisfaction	X → M (a)	.177 (.055)	.069 ~ .286	3.234	.001
Time-2 BPN satisfaction → Time-2 SWB	M → Y (b)	2.146 (.815)	.540 ~ 3.753	2.634	.009
Intervention →Time-2 SWB	Direct (c')	.667 (.664)	-.642 ~ 1.977	1.005	.316
Intervention →Time-2 SWB	Total (c)	1.048 (.657)	-.247 ~ 2.344	1.595	.112

BPN, Basic psychological needs; SWB, Subjective well-being

Bootstrap confidence intervals were estimated using 5,000 resamples.

S.14 Linear regression models examining intervention group, Time-2 competence satisfaction, and Time-2 subjective well-being in randomized trial

Independent variables	Dependent variables								
	Time-2 competence satisfaction			Time-2 Subjective well-being			Time-2 Subjective well-being		
	B (SE)	*t*	*p*	B (SE)	*t*	*p*	B (SE)	*t*	*p*
Constant	3.11 (.44)	7.10	<.001	6.72 (3.12)	2.15	.032	2.78 (3.43)	.81	.418
Intervention group	.26 (.09)	2.83	.005	1.02 (.65)	1.56	.120	.69 (.66)	1.05	.294
Time-2 competence	-	-	-	-	-	-	1.27 (.49)	2.61	.010
Control variables									
Time-1 subjective well-being	.02 (.03)	.68	.497	3.48 (.20)	17.34	<.001	3.45 (.20)	17.44	<.001
Time-1 competence	.40 (.05)	8.64	<.001	-.55 (.33)	-1.64	.102	-1.06 (.38)	-2.77	.006
Donation frequency	-.01 (.01)	-1.63	.105	-.10 (.06)	-1.72	.087	-.09 (.06)	-1.44	.150
Gender	-.06 (.10)	-.64	.526	-1.23 (.68)	-1.81	.071	-1.15 (.67)	-1.72	.087
Age	.01 (.01)	1.55	.122	.09 (.06)	1.68	.095	.08 (.06)	1.42	.159
Education level	.04 (.05)	.72	.474	-.33 (.38)	-.89	.377	-.38 (.37)	-1.03	.307
Employment status	-.02 (.14)	-.16	.873	-1.32 (.98)	-1.35	.179	-1.29 (.96)	-1.34	.182
Income	-.01 (.04)	-.24	.813	-.75 (.29)	-2.58	.010	-.74 (.29)	-2.58	.011
Marital status	-.02 (.16)	-.13	.894	2.40 (1.11)	2.16	.032	2.43 (1.1)	2.22	.028
Number of Children	-.02 (.10)	-.19	.852	-.47 (.72)	-.65	.518	-.44 (.71)	-.62	.534
Time-since-donation	-.01 (.03)	-.44	.658	-.03 (.18)	-.16	.872	-.02 (.18)	-.08	.933
Adjusted R^2	.28			.63			.64		
F	$F_{(12,210)} = 8.17, p < .001$			$F_{(12,210)} = 32.74, p < .001$			$F_{(13,209)} = 31.58, p < .001$		

N = 223

S.15 Mediation estimates of intervention on Time-2 subjective well-being through Time-2 competence satisfaction

Path	Effect	Estimate (SE)	95% (CI)	*t/z*	*p*
Intervention →Time-2 competence satisfaction → Time-2 SWB	Indirect (a × b)	.328 (.207)	.011 ~ .808	1.583	.113
Intervention →Time-2 competence satisfaction	X → M (a)	.259 (.092)	.078 ~ .440	2.825	.005
Time-2 competence satisfaction → Time-2 SWB	M → Y (b)	1.265 (.485)	.309 ~ 2.221	2.608	.010
Intervention →Time-2 SWB	Direct (c')	.691 (.657)	-.603 ~ 1.986	1.052	.294
Intervention →Time-2 SWB	Total (c)	1.019 (.653)	-.269 ~ 2.307	1.559	.120

SWB, Subjective well-being

Bootstrap confidence intervals were estimated using 5,000 resamples.
