# Supplementary material for: Blood donation and subjective wellbeing: a cross-sectional survey and a randomized trial
Source: Front Psychol. 2026 Mar 27;17:1795243. doi: 10.3389/fpsyg.2026.1795243 (PMC13065722; doi:10.3389/fpsyg.2026.1795243)
Supplement: Supplementary file 3 [file Data_Sheet_3.pdf]

Effects of the gratitude reinforcement message on Time-2 subdimension outcomes

ANCOVA analyses of the SWB and BPN subdimensions (Supplemental table 16-21) indicated that the intervention was not significantly associated with positive affect, negative affect, or life satisfaction at Time-2 (T_2). However, small but significant effects were observed for autonomy and competence satisfaction. Across all subdimensions, T_2 scores were strongly associated with their corresponding Time-1 (T_1) levels.

S.16 ANCOVA model predicting Time-2 positive affect

Predictor	*B (SE)*	*t*	*p*	*95%CI*	Partial η^2
Intercept	.84 (1.19)	.70	.485	-1.52 ~ 3.19	.00
Group					
Intervention	.48 (.34)	1.39	.165	-.20 ~ 1.15	.01
Control	reference				
Control variables					
Time-1 positive affect	2.32 (.21)	10.95	<.001	1.90 ~ 2.74	.36
Donation frequency	-.03 (.03)	-1.10	.270	-.10 ~ .03	.01
Gender	-.09 (.35)	-.26	.793	-.79 ~ .60	.00
Age	.03 (.03)	.90	.371	-.03 ~ .08	.00
Education level	.06 (.20)	.33	.742	-.32 ~ .45	.00
Employment status	-.78 (.50)	-1.58	.116	-1.76 ~ .20	.01
Income	-.27 (.15)	-1.81	.072	-.57 ~ .02	.02
Marital status	.09 (.58)	1.55	.122	- .24 ~ 2.04	.01
Number of Children	-.03 (.38)	-.07	.940	-.77 ~ .71	.00
Time-since-donation	-.07 (.09)	-.71	.479	-.25 ~ .12	.00

N = 223

Adjusted R^2 = .39

S.17 ANCOVA model predicting Time-2 negative affect

Predictor	*B (SE)*	*t*	*p*	*95%CI*	Partial η^2
Intercept	-1.98 (1.49)	-1.32	.187	-4.93 ~ .97	.01
Group					
Intervention	-.44 (.43)	-1.04	.299	-1.29 ~ .40	.01
Control	reference				
Control variables					
Time-1 negative affect	2.85 (.27)	10.70	<.001	2.33 ~ 3.38	.35
Donation frequency	.03 (.04)	.71	.481	-.05 ~ .10	.00
Gender	.20 (.44)	.45	.654	-.68 ~ 1.08	.00
Age	-.05 (.04)	-1.54	.126	-.12 ~ .02	.01
Education level	.15 (.25)	.61	.546	-.33 ~ .63	.00
Employment status	.55 (.63)	.88	.381	-.69 ~ 1.79	.00
Income	.40 (.19)	2.14	.033	.03 ~ .78	.02
Marital status	-1.06 (.72)	-1.46	.145	-2.49 ~ .37	.01
Number of Children	.91 (.47)	1.93	.055	-.02 ~ 1.84	.02
Time-since-donation	-.07 (.12)	-.57	.567	-.30 ~ .16	.00

N = 223

Adjusted R^2 = .37

S.18 ANCOVA model predicting Time-2 life satisfaction

Predictor	*B (SE)*	*t*	*p*	*95%CI*	Partial η^2
Intercept	-.66 (1.21)	-.55	.584	-3.05 ~ 1.72	.00
Group					
Intervention	.31 (.35)	.90	.370	-.37 ~ .99	.00
Control	reference				
Control variables					
Time-1 life satisfaction	3.45 (.23)	14.91	<.001	3.00 ~ 3.91	.51
Donation frequency	-.05 (.03)	-1.67	.096	-.12 ~ .01	.01
Gender	-.77 (.36)	-2.15	.033	-1.47 ~ -.06	.02
Age	.05 (.03)	1.80	.074	.00 ~ .11	.02
Education level	-.21 (.20)	-1.08	.281	-.61 ~ .18	.01
Employment status	-.92 (.51)	-1.81	.072	-1.92 ~ .08	.02
Income	.07 (.15)	.47	.642	-.23 ~ .37	.00
Marital status	.57 (.59)	.97	.332	-.59 ~ 1.73	.00
Number of Children	.43 (.38)	1.12	.264	-.33 ~ 1.18	.01
Time-since-donation	.02 (.09)	.19	.853	-.17 ~ .20	.00

N = 223

Adjusted R^2 = .58

S.19 ANCOVA model predicting Time-2 autonomy satisfaction

Predictor	*B (SE)*	*t*	*p*	*95%CI*	Partial η^2
Intercept	1.76 (.46)	3.84	<.001	.86 ~ 2.67	.07
Group					
Intervention	.18 (.05)	2.96	.003	.08 ~ .39	.04
Control	reference				
Control variables					
Time-1 autonomy	.64 (.06)	10.85	<.001	.52 ~ .76	.36
Donation frequency	-.01 (.01)	-1.42	.157	-.03 ~ .00	.01
Gender	.13 (.08)	1.57	.117	-.03 ~ .29	.01
Age	.01 (.01)	2.17	.031	.00 ~ .03	.02
Education level	.04 (.05)	.77	.444	-.06 ~ .13	.00
Employment status	-.09 (.12)	-.80	.426	-.32 ~ .14	.00
Income	.00 (.04)	.14	.888	-.06 ~ .07	.00
Marital status	-.18 (.14)	-1.28	.200	-.45 ~ .09	.01
Number of Children	-.07 (.09)	-.81	.416	-.25 ~ .10	.00
Time-since-donation	-.01 (.02)	-.37	.710	-.05 ~ .03	.00

N = 223

Adjusted $R^2 = .39$

S.20 ANCOVA model predicting Time-2 competence satisfaction

Predictor	*B (SE)*	*t*	*p*	*95%CI*	Partial η^2
Intercept	3.07 (.43)	7.09	<.001	2.21 ~ 3.92	.19
Group					
Intervention	.26 (.09)	2.84	.005	.08 ~ .44	.04
Control	reference				
Control variables					
Time-1 competence	.41 (.05)	8.87	<.001	.32 ~ .50	.27
Donation frequency	-.01 (.01)	-1.68	.095	-.03 ~ .00	.01
Gender	-.06 (.09)	-.67	.502	-.25 ~ .12	.00
Age	.01 (.01)	1.70	.090	.00 ~ .03	.01
Education level	.04 (.05)	.72	.471	-.07 ~ .14	.00
Employment status	-.05 (.13)	-.35	.725	-.31 ~ .21	.00
Income	-.01 (.04)	-.18	.858	-.09 ~ .07	.00
Marital status	-.01 (.16)	-.09	.929	-.32 ~ .29	.00
Number of Children	-.02 (.10)	-.19	.846	-.22 ~ .18	.00
Time-since-donation	-.01 (.02)	-.43	.666	-.06 ~ .04	.00

N = 223

Adjusted $R^2 = .28$

S.21 ANCOVA model predicting Time-2 relatedness satisfaction

Predictor	*B (SE)*	*t*	*p*	*95%CI*	Partial η^2
Intercept	2.56 (.52)	4.89	<.001	1.53 ~ 3.59	.10
Group					
Intervention	.03 (.10)	.26	.793	-.18 ~ .23	.00
Control	reference				
Control variables					
Time-1 relatedness	.57 (.08)	7.15	<.001	.41 ~ .72	.19
Donation frequency	.00 (.01)	.47	.640	-.01 ~ .02	.00
Gender	-.11 (.11)	-1.05	.293	-.32 ~ .10	.01
Age	-.02 (.01)	-1.87	.063	-.03 ~ .00	.02
Education level	-.03 (.06)	-.52	.602	-.15 ~ .09	.00
Employment status	.11 (.15)	.74	.463	-.18 ~ .40	.00
Income	-.02 (.05)	-.47	.638	-.11 ~ .07	.00
Marital status	-.12 (.18)	-.70	.484	-.47 ~ .22	.00
Number of Children	.12 (.11)	1.09	.276	-.10 ~ .35	.01
Time-since-donation	.00 (.03)	-.13	.896	-.06 ~ .05	.00

N = 223

Adjusted $R^2 = .19$

Exploratory within-group changes from Time-1 to Time-2

Paired-samples *t*-tests were conducted to examine within-group changes between T_1 and T_2. These analyses are presented for descriptive purposes and should not be interpreted as causal estimates of the intervention effect.

Within the Intervention group, both SWB and BPN satisfaction increased significantly from T_1 to T_2, whereas no significant changes were observed in the Control group (Supplemental table 22). Analyses of the subdimensions further indicated higher positive affect, lower negative affect, and increased competence satisfaction at T_2 within the Intervention group (Supplemental table 23).

S.22 Paired-samples *t*-test results of Time-1 and Time-2 subjective well-being and basic psychological needs satisfaction

	Intervention (*N* = 104)	*p*	Control (*N* = 119)	*p*
	Difference score		*Difference score*	
Time-1→Time-2 Subjective well-being	2.17	.001	.97	.126
Time-1→Time-2 Basic psychological needs	.18	.001	.00	.939

S.23 Paired-samples *t*-test results of other variables

	Intervention (*N* = 104)	*p*	Control (*N* = 119)	*p*
	Difference score		*Difference score*	
Time-1→Time-2 Positive affect	.64	.010	.07	.806
Time-1→Time-2 Negative affect	-1.02	.001	- .64	.066
Time-1→Time-2 Life satisfaction	.52	.127	.16	.606
Time-1→Time-2 Autonomy	.10	.231	- .13	<.001
Time-1→Time-2 Competence	.35	.004	.03	.417
Time-1→Time-2 Relatedness	.10	.349	.09	.030

Comparisons of Time-2 variables in randomized trial and Time-1 variables in baseline survey

Supplemental table 24 presents that T_2 SWB and BPN satisfaction in Intervention group were significantly higher than T_1 measures in both Recall and Non-recall groups; whereas no significant differences were found between T_2 SWB and BPN satisfaction in Control group and T_1 values from neither Recall nor Non-recall group.

Supplemental table 25 shows that positive affect in the Intervention group at T_2 was significantly higher than in the Recall group at T_1, while negative affect in the Intervention group was lower. Additionally, autonomy and competence satisfaction were both significantly higher in the Intervention group at T_2 compared to T_1 values in the Recall and Non-recall groups. Similarly, T_2 autonomy and competence satisfaction in the Control group were also higher than T_1 values in the Non-recall group.

S.24 Independent-sample *t*-test results of comparing Time-2 subjective well-being and basic psychological needs in Donation group to Time-1 subjective well-being and basic psychological needs in Recall group and Non-recall group

Comparison	Subjective well-being	Basic psychological needs
	p (t)	*p (t)*
Intervention *vs.* Recall	.029 (2.19)	.009 (2.64)
Intervention *vs.* Non-recall	<.001 (3.53)	<.001 (4.07)
Control *vs.* Recall	.430 (.79)	.971 (.04)
Control *vs.* Non-recall	.100 (1.65)	.166 (1.39)

$N_{(Intervention)} = 104$; $N_{(control)} = 119$; $N_{(Recall)} = 517$; $N_{(Non\text{-}recall)} = 526$

S.25 Independent-sample *t*-test results of other Time-2 and Time-1 factors among groups

Comparison	Positive affect	Negative affect
	p (t)	*p (t)*
Intervention *vs.* Recall	.112 (1.59)	.107 (-1.61)
Intervention *vs.* Non-recall	.002 (3.13)	.042 (-2.04)
Control *vs.* Recall	.710 (-.37)	.463 (-.73)
Control *vs.* Non-recall	.186 (1.32)	.234 (-1.19)
**Comparison**	**Life satisfaction**	**Autonomy**
	p (t)	*p (t)*
Intervention *vs.* Recall	.090 (1.70)	<.001 (3.55)
Intervention *vs.* Non-recall	.315 (1.01)	<.001 (5.35)
Control *vs.* Recall	.357 (.92)	.564 (.38)
Control *vs.* Non-recall	.862 (.17)	.023 (2.28)
**Comparison**	**Competence**	**Relatedness**
	p (t)	*p (t)*
Intervention *vs.* Recall	.001 (3.21)	.921 (-.01)
Intervention *vs.* Non-recall	<.001 (5.63)	.893 (.14)
Control *vs.* Recall	.775 (.29)	.570 (-.57)
Control *vs.* Non-recall	.003 (2.98)	.670 (-.43)

$N_{(Intervention)} = 104$; $N_{(control)} = 119$; $N_{(Recall)} = 517$; $N_{(Non\text{-}recall)} = 526$

Time-2 Blood donation intention and donation behavior

When donors made a subsequent donation, the Guangzhou Blood Center's system automatically sent an SMS confirmation immediately after completion. The principal investigator verified re-donation through the Center's database, and each participant was followed for 365 days. A 1-year follow-up period was chosen to allow sufficient opportunity for repeat donation, given that Mainland China requires a minimum 180-day interval between whole-blood donations (and 90 days for platelet apheresis after whole-blood donation).

For the intention-to-treat analysis, the re-donation rate was calculated as the proportion of all initially randomized donors who donated again within the follow-up period, regardless of whether they completed the T_2 questionnaire. A per-protocol analysis was also conducted, in which re-donation rates were calculated only among donors who completed the T_2 assessment.

T_2 Blood donation intention. An ANCOVA model was conducted to examine predictors of T_2 blood donation intention while controlling for baseline variables and time-since-donation (Supplemental table 26). The intervention was not significantly associated with T_2 intention (*B (SE)* = −.01 (.10), p = .934). T_1 blood donation intention was positively associated with T_2 intention (*B (SE)* = .20 (.06), p = .001, partial η^2 = .05). In addition, time-since-donation showed a small negative association with T_2 intention (*B (SE)* = −.06 (.03), p = .021, partial η^2 = .03). No significant associations were observed for SWB or BPN at either T_1 or T_2.

In addition, paired-samples *t*-tests showed no significant within-group increases from T_1 to T_2 (Intervention: $p_{T1\rightarrow T2} = .256$, Control: $p_{T1\rightarrow T2} = .439$).

Re-donation behavior. Within one year, 259 donors (43.1%) made at least one additional donation. The average interval between completion of the second questionnaire and re-donation was 244.5 ± 79.0 days (range: 84-361 days), with no significant difference in re-donation rates between the two groups (Supplemental table 27). Logistic regression using stepwise forward selection (Wald) showed that donation frequency strongly predicted re-donation behavior, whereas T_2 SWB was negatively associated with re-donation (Supplemental table 28). Re-donation behavior was not significantly related to group assignment, blood donation intention (T_1 or T_2), or BPN satisfaction (T_1 or T_2).

S.26 ANCOVA model predicting Time-2 blood donation intention

Predictor	*B (SE)*	*t*	*p*	*95%CI*	Partial η^2
Intercept	4.64 (.68)	6.77	.000	3.29 ~ 5.99	.18
Group				~	
Intervention	-.01 (.10)	-.08	.934	-.20 ~ .18	.00
Control					
Control variables					
Time-1 blood donation intention	.20 (.06)	3.25	.001	.08 ~ .33	.05
Time-1 subjective well-being	.02 (.05)	.46	.643	-.07 ~ .11	.00
Time-1 basic psychological needs	.23 (.13)	1.71	.089	-.04 ~ .50	.01
Time-2 subjective well-being	.00 (.01)	.28	.778	-.02 ~ .02	.00
Time-2 basic psychological needs	-.04 (.12)	-.35	.725	-.28 ~ .19	.00
Donation frequency	.01 (.01)	1.63	.106	.00 ~ .03	.01
Gender	-.08 (.10)	-.84	.404	-.27 ~ .11	.00
Age	.00 (.01)	.10	.924	-.01 ~ .02	.00
Education level	-.03 (.05)	-.54	.593	-.13 ~ .08	.00
Employment status	-.10 (.14)	-.69	.488	-.37 ~ .18	.00
Income	-.02 (.04)	-.45	.655	-.10 ~ .06	.00
Marital status	.11 (.16)	.68	.499	-.21 ~ .43	.00
Number of Children	.03 (.10)	.25	.805	-.18 ~ .23	.00
Time-since-donation	-.06 (.03)	-2.32	.021	-.11 ~ -.01	.03

N = 223

Adjusted $R^2 = .15$

S.27 Comparisons of re-donation behavior within 1-year follow-up by intention-to-treat and per-protocol analyses

Analysis	Groups	*N*	Actual blood donation rate (n, %)	Raw *p* (χ^2)	Bonferroni corrected *p* (χ^2)
Intention-to-treat	Intervention	300	133 (44.3)	.540 (.37)	.596 (.28)
	Control	301	126 (41.9)		
Per-protocol	Intervention	104	51 (49.0)	.648 (.21)	.747 (.10)
	Control	119	61 (52.1)		

S.28 Stepwise forward-variable selection (Wald) Logistic regression models predicting re-donation behavior in the randomized trial

Models		*B (SE)*	*Wald*	*p*	*Exp (B)*
1	Constant	-.82 (.22)	14.06	<.001	.44
	Donation frequency	.16 (.04)	20.35	<.001	1.18
2	Constant	-.78 (.22)	12.38	<.001	.46
	Donation frequency	.16 (.04)	21.12	<.001	1.18
	Time-2 Subjective well-being	-.04 (.02)	4.36	.037	.96

N = 223

Control variables included:

Time-1 and Time-2: blood donation intention, subjective well-being, basic psychological needs satisfaction;

Donation frequency, gender, age, education level, employment status, income, marital status, number of children and time-since-donation.
